# Capillaroscopy in Psoriatic and Rheumatoid Arthritis: A Useful Tool for Differential Diagnosis

**DOI:** 10.1155/2013/957480

**Published:** 2013-12-12

**Authors:** Dario Graceffa, Beatrice Amorosi, Elisa Maiani, Claudio Bonifati, Maria Sole Chimenti, Roberto Perricone, Aldo Di Carlo

**Affiliations:** ^1^Centre for the Study and Treatment of Psoriasis, Department of Clinical Dermatology, San Gallicano Dermatological Institute (IRCCS), Rome, Italy; ^2^Rheumatology, Allergology and Clinical Immunology, Department of Internal Medicine, University of Rome Tor Vergata, Rome, Italy; ^3^San Gallicano Dermatological Institute, Rome, Italy

## Abstract

Impairment of capillaries permeability and changes of microcirculation are associated with inflammatory arthritis. In order to demonstrate microvascular differences between psoriatic arthritis (PsA) and rheumatoid arthritis (RA) we analyzed capillaroscopic abnormalities such as megacapillaries, haemorrhages, ramifications, and avascular areas in patients affected by these two rheumatic disorders. Moreover to identify specific capillaroscopy patterns we analyzed the following parameters: venous limb diameter, arterial limb diameter, capillary loop diameter, amplitude of the capillary loop, linear density of capillaries (on 2 mm), and number of twisted capillaries (on 4 mm). Through a comparative morphometric analysis of capillaroscopy, our study demonstrated the presence of specific microvascular differences between PsA and RA providing an additional diagnostic tool for the differential diagnosis. We also suggest that capillaries structural abnormalities might reflect endothelial injury due to systemic inflammation during chronic arthritis.

## 1. Introduction

Psoriatic arthritis (PsA) is a chronic inflammatory disease affecting 0.04%–0.1% of the general population and occurs in one-third of patients with psoriasis. PsA can lead to severe bone erosions and joints destruction resulting in physical disability [1].

Nowadays, no specific laboratory tests are available for PsA; it is not always possible, therefore, confirm the diagnosis and provide an objective prognosis, especially at an early stage of disease.

The differential diagnosis between PsA and RA is very difficult in PsA patients with slight cutaneous signs and rheumatoid-like joint involvement [2–4]. Presence of citrullinated peptide antibodies has a high specificity for RA, but, among that, they are rarely seen in PsA patients [5].

Morphological and rheological changes in microcirculation have been widely demonstrated in PsA and RA patients.

Impairment of capillaries permeability and alterations in the connections between endothelium and extracellular matrix characterize these damages, resulting in abnormal structure and shape of the capillaries in the dermal papillae [6–8].

Capillaroscopy allows the study “*in vivo*” of morphological and functional characteristics of microcirculation. Several authors have tried to identify specific microvascular characteristics of cutaneous psoriasis and PsA; these studies were mainly conducted on the skin plaques, on synovial membrane and at nailfold level. However, the results are not univocal [9–12].

Aim of our study was to demonstrate specific microvascular differences between PsA and RA, evaluable by nailfold capillaroscopy.

We considered capillaroscopy of the psoriatic plaque not suitable for our purpose for the following reason.

Microscopic observation of psoriatic plaque allows a frontal view of capillaries; it is therefore very difficult to establish if is displayed a single capillary or a branch of another. This artefact is particularly relevant in course of PsA where capillaries are typically branched and tortuous. It may cause a loss of accuracy in estimating capillaries density; conversely nailfold capillaroscopy providing a longitudinal view of capillary allows to follow it for the entire length.

## 2. Patients and Methods

From January 2012 to March 2013, 30 patients affected by PsA, 30 patients affected by RA, 30 patients affected by Psoriasis (Pso) without signs of arthropathy, and 30 healthy subjects as control, attending the Rheumatology Department of the University of Rome “Tor Vergata” and the Dermatology Department of San Gallicano Dermatologica Institute, were enrolled.

Diagnosis of PsA and RA was made, respectively, according to the clASsification Criteria for Psoriatic Arthritis (CASPAR) and the American College of Rheumatology criteria (ACR) [13, 14]. Informed consent was obtained before capillaroscopy evaluation.

Inclusion and exclusion criteria used are summarized in Table 1.

Patients and healthy subjects were comparable for age, sex, main cardiovascular risk factors and other vascular diseases (*P*-values = N.S.). Patients treatments performed were equivalent in all groups. Patients demographic and clinical characteristics are summarized in Table 2.

Nailfold capillaroscopy was performed using a videomicroscope “Alpha Strumenti Srl, Melzo Italy” with optic probes equipped with 100x and 200x magnification contact lenses.

### 2.1. Morphological Study

We analyzed the principal vascular abnormalities (megacapillaries, haemorrhages, ramifications, and avascular areas) using a semiquantitative rating scale to score these changes, according to previous studies [11].

### 2.2. Morphometric Study (Figure 1)

We measured the following parameters: venous limb diameter (*μ*m), arterial limb diameter (*μ*m), capillary loop diameter (*μ*m), amplitude of the capillary loop (*μ*m), linear density of capillaries expressed as number of loops on 2 mm, and number of twisted capillaries (on 4 mm).

It was not possible to perform reliable assessments of the capillary length due to high tortuosity and difficulties in determining the angle between capillary and skin surface, especially in psoriatic patients.

Data were statistically analyzed with *GraphPad Prism 5 statistical software (GraphPad Software, San Diego, CA)* and expressed as mean ± SD. Significance of the data obtained was assessed with Student's *t*-test (statistical significance was set at *P* ≤ 0.05).

## 3. Results

### 3.1. Morphological Study

Principal capillaroscopic abnormalities (megacapillaries, haemorrhages, ramifications, and avascular areas) resulted similar in Pso, PsA and RA patients (*P*-values = N.S.).

### 3.2. Morphometric Study

Capillaries of patients with Pso, PsA, and RA showed, respectively, a venous limb diameter of 17.6 *μ*m ± 5.2, 18 *μ*m ± 5 and 22 *μ*m ± 4 and an arterial limb diameter of 15.3 *μ*m ± 4.3, 15 *μ*m ± 3, and 16 *μ*m ± 4. Capillary loop diameter was 26.8 *μ*m ± 6.3 in Pso, 27 *μ*m ± 7 in PsA and equal to 30 *μ*m ± 8 in RA patients. The capillary loop amplitude was of 42.2 *μ*m ± 10.3 in Pso, 56 *μ*m ± 12 in RA, 40 *μ*m ± 9 in PsA. Linear density of capillaries was 13.2 ± 2.1 in Pso, 13 ± 1 in PsA, and 16 ± 2 in RA.

The number of twisted capillaries on 4 mm was of 10.5 ± 2.1 in Pso, 12.2 ± 3.2 in PsA, and 7.1 ± 1.2 in RA patients. Comparative analysis of patients affected by Pso and PsA, with the control group showed significant statistical differences in three of considered parameters (amplitude of the capillary loop, linear density of capillaries, and number of twisted capillaries) with no differences between Pso and PsA. Capillaries of RA patients showed significant statistical differences if compared with the control group in venous limb diameter, arterial limb diameter, capillary loop diameter, and amplitude of the capillary loop.

The study showed, furthermore, significant statistical differences between RA patients and psoriatic patients (with no differences between Pso and PsA) in all considered parameters.

Results are summarized in Table 3.

## 4. Discussion

Activation of vascular endothelium play a key role in initiation and progression of systemic inflammatory disease as psoriasis, psoriatic arthritis and rheumatoid arthritis. Deregulation of intercellular adhesion molecule-1 (ICAM-1) and vascular adhesion molecule-1 (VCAM-1) was detected in the skin and synovial membrane of patients with psoriasis and psoriatic arthritis [15].

Moreover, levels of VEGF (Vascular Endothelial Growth Factor) and sICAM (Soluble Intracellular Adhesion Molecules), considered to be biomarkers of vascular injury, have been found altered in course of chronic arthritis. There are controversial data about the role of VEGF in course of psoriasis and psoriatic arthritis. Experimental models of its inhibition seem to reduce inflammation; on the other hand the human use of Bevacizumab, a humanized monoclonal antibody that inhibits VEGF-A, did not show positive effects on psoriasis and there are case reports of reactivation of PSO and PsA in course of treatment.

In addition, VEGF and molecules membership are closely associated with the activity of key inflammatory molecules involved in psoriasis such as TNF and NFKB [16, 17].

Significant decrease of MMP-9 and MMP-2 levels in the sera was associated with clinical improvement and with the decrease of TNF-*α*, VEGF, and E-selectin, angiogenic molecules already known to be implicated in clinical expression of psoriasis [18].

In the present study, morphometric analysis showed statistically significant differences between PsA, Pso, and RA.

In RA, measures of the venous limb diameter (efferent branch of the capillary loop), arterial limb diameter (afferent branch of the capillary loop), and loop diameter were significantly higher than in PsA, Pso, and the control group. The trend toward expansion, found in the capillaries of RA patients, is more evident at the efferent branch of the loop and may represent an early manifestation of vascular damage in the microcirculation [19].

In 1994, Hachulla et al., showed microvascular permeability alterations in RA, to confirming the existence of a microangiopathy [7].

In addition, Meyer et al. showed modifications of the normal blood flow velocity and microvascular dysfunction in RA [20].

Zaric et al., in a study conducted in patients with RA and PsA, reported capillaroscopic differences between the two diseases [8]. However, the differences concerned only the linear density of capillaries and measurements of the average diameter of the capillary segments of the loop were not carried out.

A study conducted on PsA patients, in agreement with our observations, showed a reduction in capillaries linear density [6].

It can be hypothesized that the density of the capillaries is reduced in patients suffering from psoriasis as a result of changes in the normal angiogenesis. On the other hand the loss of normal binding between the endothelial cell and matrix of the papillary dermis could explain the capillary branching and tortousity we detected in these patients.

The finding of a reduction of density in psoriatic patients is partially in contrast with those reported in studies conducted on the psoriatic plaque where a slight increase of capillary density was detected [21].

We believe that this observation is directly linked to the changes of the capillary in course of psoriasis (tendency to terminal tortuosity and branching).

During microscopic observation of psoriasis plaque, indeed, capillaries are shown frontally so it is possible that ramifications and tortuosity (typically associated with psoriasis) are incorrectly interpreted as individual capillaries rather than ramifications of the same capillary. This artefact is absent in the periungual study because the longitudinal view allows to follow the entire course of capillary within the dermal papilla.

In our opinion, the increase in the perfusion of the plaque is connected to a vasodilation and an increase of the flow velocity rather than to an increased density of capillaries.

**Figure 2:**
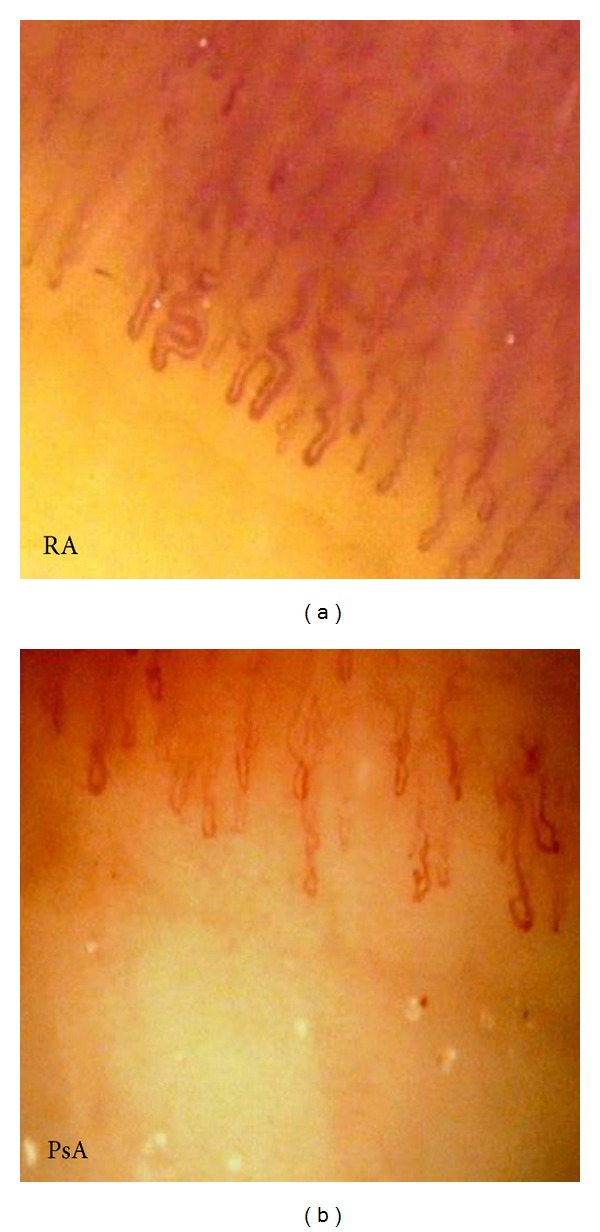
Capillaroscopic images of patients with RA and PsA. PsA patient has a higher number of twisted capillaries and a lower density if compared with RA patient.

In conclusion, in RA patients we found capillaries with larger diameters if compared with those of psoriatic patients and the control group. We also observed a reduction in the linear density of the capillaries and an increased tortuosity in psoriatic patients than the other two groups. These morphologic characteristics may reflect pathogenetic differences between the two diseases providing an additional approach to establish a correct differential diagnosis (Figure 2).

In our opinion capillaroscopy can be considered a valid technique in inflammatory joint diseases to analyze microvascular circulation.

## Figures and Tables

**Figure 1:**
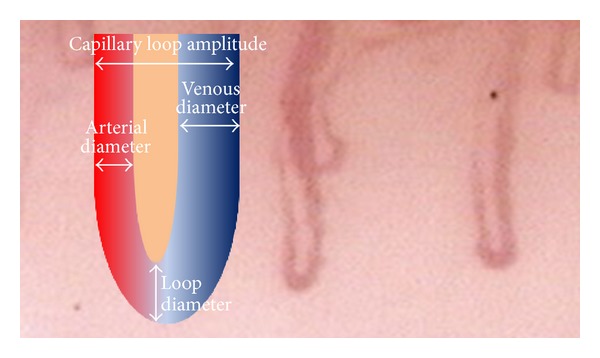
Capillary dimensions measured in the morphometric study.

**Table 1:** Inclusion and exclusion criteria for participation in the study.

Inclusion criteria:	
(i) Psoriasis vulgaris moderate-severe (Psoriasis Area Severity Index) PASI > 6	
(ii) Psoriatic arthritis diagnosed according to Classification Criteria for Psoriatic Arthritis (CASPAR)	
(iii) Rheumatoid arthritis diagnosed according to American College of Rheumatology criteria (ACR)	
Exclusion criteria:	
(i) Nail psoriasis	
(ii) Primary or secondary Raynaud's phenomenon	
(iii) Acrocyanosis	
(iv) Cardiovascular risk factors and/or coagulopathy	
(v) Vasoactive therapies and/or anticoagulants	

**Table 2:** Patients demographic and clinical characteristics.

	Pso	PsA	RA	Controls
Patients (number)	30	30	30	30
Gender (male/female)	14/16	11/19	12/18	15/15
Age	48.1 ± 12.3	51.2 ± 13.2	53.1 ± 13.6	47.9 ± 12.6
PASI score	11.1 ± 3.1	9.6 ± 2.8	0	0
DAS-28 ESR	0	4.1 ± 1.9	4.9 ± 2.2	0
CyA tp	11/30	3/30	2/30	0
MTX tp	6/30	15/30	19/30	0
Disease duration	19.7 ± 4.1	11.6 ± 3.3	10.7 ± 4	0

Data are expressed as mean ± SD; PASI: psoriasis area severity index; DAS28-ESR: 28-joint disease activity score; CyA: cyclosporine A; MTX: methotrexate.

**Table 3:** Morphometric study results.

	Venous limb diameter	Arterial limb diameter	Loop diameter	Loop amplitude	Linear density (Loops/2 mm)	Tortuosity (Twisted capillaries/4 mm)
Pso	17.6 *μ*m ± 5.2	15.3 *μ*m ± 4.3	26.8 *μ*m ± 6.3	42.2 *μ*m ± 10.3	13.2 ± 2.1	10.5 ± 2.1
PsA	18 *μ*m ± 5.4	15.1 *μ*m ± 3	27.2 *μ*m ± 7.1	40.4 *μ*m ± 9.1	13.1 ± 1	12.2 ± 3.2
RA	22 *μ*m ± 4.1	16.2 *μ*m ± 4.1	30 *μ*m ± 8	56 *µ*m ± 12	16 ± 2	7.1 ± 1.2
Controls	17.5 *μ*m ± 3.1	14 *μ*m ± 3.2	26.4 *μ*m ± 5	46.1 *μ*m ± 10	16.4 ± 2.6	6.2 ± 1.1

Pso versus controls	N.S.	N.S.	N.S.	*P* < 0.001	*P* < 0.001	*P* < 0.001
PsA versus controls	N.S.	N.S.	N.S.	*P* < 0.001	*P* < 0.001	*P* < 0.001
RA versus controls	*P* < 0.001	*P* < 0.001	*P* < 0.001	*P* < 0.001	N.S	N.S.
PsA versus RA	*P* < 0.001	*P* < 0.05	*P* < 0.001	*P* < 0.001	*P* < 0.001	*P* < 0.001
Pso versus RA	*P* < 0.001	*P* < 0.05	*P* < 0.001	*P* < 0.05	*P* < 0.001	*P* < 0.001
Pso versus PsA	N.S.	N.S.	N.S.	N.S.	N.S.	N.S.

Data are expressed as mean ± SD.

Statistical significance was assessed with Student's *t*-test; significance was set at *P* < 0.05.
